# Neuroinflammation and amyloid-beta 40 are associated with reduced serotonin transporter (SERT) activity in a transgenic model of familial Alzheimer’s disease

**DOI:** 10.1186/s13195-019-0491-2

**Published:** 2019-05-01

**Authors:** Athanasios Metaxas, Marco Anzalone, Ramanan Vaitheeswaran, Sussanne Petersen, Anne M. Landau, Bente Finsen

**Affiliations:** 1 Department of Neurobiology, Institute of Molecular Medicine, University of Southern Denmark, J.B. Winsløws Vej 25, DK-5000 Odense C, Denmark; 2 Department of Nuclear Medicine & PET Center, Aarhus University and Hospital, Nørrebrogade 44, Building 10G, DK-8000 Aarhus, Denmark; 3 Translational Neuropsychiatry Unit, Department of Clinical Medicine, Aarhus University, Skovagervej 2, DK-8240 Risskov, Denmark

**Keywords:** Alzheimer’s disease, *APP*_swe_*/PS1*_dE9_, Amyloid-beta, SSRIs, Serotonin transporter, DASB, Neuroinflammation, TSPO, Cytokines

## Abstract

**Background:**

Discrepant and often contradictory results have accumulated regarding the antidepressant and pro-cognitive effects of serotonin transporter (SERT) antagonists in Alzheimer’s disease.

**Methods:**

To address the discrepancy, we measured the activity and density of SERT in the neocortex of 3–24-month-old *APP*_swe_*/PS1*_dE9_ and wild-type littermate mice, by using [^3^H]DASB autoradiography and the [^3^H]5-HT uptake assay. Levels of soluble amyloid-β (Aβ), and pro-inflammatory cytokines that can regulate SERT function, such as interleukin-1β (IL-1β), interleukin-6 (IL-6), and tumor necrosis factor (TNF), were measured in parallel. Neuroinflammation in aging *APP*_swe_*/PS1*_dE9_ mice was further evaluated by [^3^H]PK11195 autoradiography.

**Results:**

Decreased SERT density was observed in the parietal and frontal cortex of 18–24-month-old *APP*_swe_*/PS1*_dE9_ mice, compared to age-matched, wild-type animals. The maximal velocity uptake rate (*V*_max_) of [^3^H]5-HT was reduced in neocortical preparations from 20-month-old transgenic vs. wild-type mice. The reduction was observed when the proportion of soluble Aβ_40_ in the Aβ_40/42_ ratio increased in the aged transgenic brain. At concentrations compatible with those measured in 20-month-old *APP*_swe_*/PS1*_dE9_ mice, synthetic human Aβ_40_, but not Aβ_42_, reduced the baseline *V*_max_ of [^3^H]5-HT by ~ 20%. Neuroinflammation in *APP*_swe_*/PS1*_dE9_ vs. wild-type mice was evidenced by elevated [^3^H]PK11195 binding levels and increased concentration of IL-1β protein, which preceded the reductions in neocortical SERT density and activity. Age-induced increases in the levels of IL-1β, IL-6, and TNF were observed in both transgenic and wild-type animals.

**Conclusions:**

The progression of cerebral amyloidosis is associated with neuroinflammation and decreased presynaptic markers of serotonergic integrity and activity. The Aβ_40_-induced reduction in the uptake kinetics of [^3^H]5-HT suggests that the activity of SERT, and potentially the effects of SERT antagonism, depend on the levels of interstitial Aβ_40_.

## Background

The discussion surrounding the effectiveness of selective serotonin reuptake inhibitors (SSRIs) in Alzheimer’s disease (AD) and related dementias has extended beyond the drugs’ effects on comorbid depression [1]. Clinical studies on the incidence of dementia have reported an increased risk of cognitive impairment following SSRI treatment [2], but also beneficial delays in the conversion from mild cognitive impairment to AD [3]. In preclinical studies, the chronic administration of SSRIs to transgenic models of AD may improve cognitive performance [4, 5], but also increases mortality rates compared to vehicle administration [6, 7]. The reasons for the heterogeneity in the antidepressant and cognitive effects of SSRIs in AD remain unknown.

The serotonin transporter (SERT) is the primary target of SSRI treatment [8]. By blocking SERT, SSRIs inhibit the clearance of extracellular serotonin (5-HT) and potentiate serotonergic neurotransmission. There is evidence to suggest that the brain levels of SERT change dynamically with AD progression. The binding of [^3^H]escitalopram is higher than control levels in the cortex and hippocampus of 4-month-old *APP*_swe_/*PS1*_dE9_ mice [9], a model of familial AD harboring mutations in the amyloid precursor protein (*APP*) and presenilin-1 (*PSEN1*; *PS1*) genes [10]. In the hippocampus of 3xTg-AD mice, a model of combined amyloidosis and tauopathy, elevated SERT immunoreactivity has been observed by 3 months of age, i.e., prior to the manifestation of amyloid-β (Aβ)-induced memory impairment or the deposition of plaques [11, 12]. These “early” increases in the density of SERT, which may decrease the level of extracellular 5-HT, contrast the degenerative reductions in the levels of several serotonergic components, including SERT, which are typically observed in the brain of aged amyloidosis models [13, 14] and advanced cases of AD [15]. The aforementioned literature implies that a low serotonergic tone may be associated with both increased and decreased SERT-binding levels, according to disease stage. Thus, it is plausible that the therapeutic efficacy of SSRIs will vary considerably during the course of AD, depending on the density and/or activity of the SERT.

Chronic, low-grade neuroinflammation constitutes hallmark AD pathology [16]. Neuroinflammation has been demonstrated in the brain of living demented patients and models of AD by molecular imaging of the translocator protein (TSPO), which is primarily a marker of microgliosis [17]. The neuroinflammatory reaction is characterized by increased microglial production of inflammatory cytokines, such as interleukin-1 beta (IL-1β) and tumor necrosis factor [TNF [18, 19]]. Moreover, IL-1β, TNF, and interleukin-6 (IL-6) are elevated in the periphery of AD patients compared to non-AD subjects [20]. Interestingly, these pro-inflammatory cytokines have been reported to alter SERT function in a cytokine-specific manner. In the mouse midbrain and striatum, IL-1β can stimulate SERT activity, by decreasing the apparent Michaelis constant (*K*_m_) of the transporter for 5-HT [21]. In neuronal cell lines and mouse synaptosomal preparations, TNF decreases *K*_m_ and increases the apparent maximal velocity rate (*V*_max_) of 5-HT uptake [22]. Unlike the stimulating effects of IL-1β and TNF, treatment with IL-6 reduces the mRNA and protein levels of SERT in the mouse hippocampus [23]. These data are indicative of a cytokine-induced mechanism for the dysregulation of SERT, which could interfere with serotonergic neurotransmission during the course of AD.

In the present study, SERT density and function were evaluated in parallel with markers of neuroinflammation in the brain of aging *APP*_swe_/*PS1*_dE9_ mice. It was hypothesized that the progression of amyloidosis would be associated with age-related alterations in the binding levels of SERT, which could be attributed to increased levels of specific neuroinflammatory markers.

## Materials and methods

### Animals

A total of 113 *APP*_swe_/*PS1*_dE9_ transgenic (TG) and littermate wild-type (WT) control mice were used in this study (109 females; 4 males). The animals were bred and maintained on a C57BL/6J background in the Biomedical Laboratory of the University of Southern Denmark. Mice were group-housed in a temperature (21 ± 1 °C) and humidity (45–65%) controlled environment, under a 12/12-h light/dark cycle (lights on: 7 am), with free access to food and water.

### [^3^H]DASB and [^3^H]PK11195 autoradiography

Animals were euthanized by cervical dislocation at 3, 6, 12, 18, and 24 months of age (*n* = 6/group, total number = 60). The brains were immediately removed, placed on a petri dish containing ice, and bisected along the midline. The brainstem from the left hemisphere was frozen on dry ice and used for measuring *Scl6a4* (*Sert)* mRNA levels by reverse transcription quantitative real-time polymerase chain reaction (RT-qPCR). The right brain hemisphere was immersed for 2 min in isopentane on dry ice (− 30 °C) and used for autoradiography. Consecutive, 20-μm-thick sagittal sections were collected on ice-cold SUPERFROST™ slides at 300 μm intervals, by using a Leica CM3050S cryostat (Nussloch, Germany). The sections were dried overnight at 4 °C in a box containing silica gel and stored at − 80 °C until use.

[^3^H]DASB autoradiography was used to measure the density of SERT, as described previously [24]. Sections were thawed at room temperature (RT) and prewashed in 50-mM Tris-HCl buffer (pH 7.4), containing 150 mM NaCl and 5 mM KCl (3 × 15 min). The sections were subsequently incubated for 2 h in the same buffer, containing 1 nM [^3^H]DASB (specific activity 80 Ci/mmol; ART1523, American Radiolabeled Chemicals, Inc.). To determine non-specific binding (NSB), adjacent sections were radiolabeled with 1 nM [^3^H]DASB in the presence of 10 μM paroxetine HCl hemihydrate (P9623; Sigma-Aldrich). Incubations were terminated by three 1-min washes into ice-cold 50-mM Tris-HCL buffer (pH 7.4), followed by a rapid rinse in ice-cold deionized H_2_O (dH_2_O, Ultra-Clear; Siemens).

[^3^H]PK11195 autoradiography was used to measure the density of the neuroinflammatory marker TSPO [25]. Sections were thawed at RT and prewashed in 50-mM Tris-HCl buffer (pH 7.4), containing 150 mM NaCl, 5 mM KCl, 1.5 mM MgCl_2_, and 1.5 mM CaCl_2_. Incubations were carried out for 2 h in the same buffer, containing 3 nM [^3^H]PK11195 (specific activity 82.7 Ci/mmol; NET885, PerkinElmer). NSB was determined in the presence of 10 μM unlabeled PK11195 (C0424; Sigma-Aldrich). The incubations were terminated as described for [^3^H]DASB.

The labeled sections were dried for 2 h under a stream of cold air and placed against Carestream® Kodak® BioMax MR film for a period of 25 days. To allow quantification, ^3^H microscales of known radioactive concentration were also apposed to each film (American Radiolabeled Chemicals, Inc.). After development with KODAK substitute D-19 developer (TED PELLA, Inc.), images were digitized using a white sample tray and the Coomassie Blue settings on a ChemiDoc™ MP imaging system (BIO-RAD). Values of specific binding were calculated by subtracting the level of non-specific from total binding levels, using ImageJ software (v1.50e; National Institutes of Health, MD, USA). For each animal, specific binding in the frontal, parietal, occipital, and temporal cortices was analyzed on 2–6 consecutive sections, by reference to the Allen Mouse Brain Atlas for sagittal sections [26].

### Sert RT-qPCR

Trizol®-isolated RNA (2 μg) from the brainstem of 3-, 6-, 12-, 18-, and 24-month-old TG and WT mice was reverse-transcribed to cDNA with the Applied Biosystems™ high-capacity cDNA transcription kit (Thermo Fisher Scientific). Samples of 20 μL were run in triplicate on a StepOne-Plus™ Real-Time PCR system (Applied Biosystems™, Thermo Fisher Scientific). Each sample contained nuclease-free H_2_O (Thermo Fisher Scientific), 1× Maxima SYBR green/probe master mix (Thermo Fisher Scientific), 500 nM forward and reverse primers (TAG Copenhagen A/S), 5× diluted cDNA for *Sert*, and 10× diluted cDNA for hypoxanthine phosphoribosyltransferase (*Hprt*), which was used as a reference gene. Primer sequences for *Hprt* and *Sert* have been described previously [24, 27]. After normalization to *Hprt*, data were expressed as fold-change from the mean *Sert*/*Hprt* ratio of 3-month-old WT mice. Nuclease-free H_2_O and genomic DNA instead of cDNA were used to control for contamination.

### [^3^H]5-HT transport assay

The assay was performed as detailed previously [22], using freshly isolated cortical tissue from a female, 3-, 6-, 12-, and 20-month-old, WT and TG mice (*n* = 5–7/group, total number = 49). Freshly prepared homogenates were required for the assay, as preparations of frozen material led to undetectable levels of specific binding.

Cortices were homogenized in 10 vol (*w*/*v*) of ice-cold HEPES (10 mM)/sucrose (0.32 M) buffer (pH 7.4), by using a motor-driven, Teflon-glass tissue grinder at 800 rpm (Wheaton Instruments). The homogenate was centrifuged for 10 min at 1000×*g* in a refrigerated centrifuge (4 °C; RC M150 GX, Sorvall). The pellet was discarded, and 200 μL of the supernatant (S1) kept on ice for determining the levels of IL-1β, IL-6, TNF, and Aβ_40/42_ by Meso Scale Discovery immunoassays. The S1 supernatant was transferred to clean tubes and centrifuged at 100,000×*g* for 20 min (4 °C). The resulting pellet was suspended in 10 mM HEPES buffer (pH 7.4), containing 130 mM NaCl, 1.3 mM KCl, 2.2 mM CaCl_2_, 1.2 mM MgSO_4_, 1.2 mM KH_2_PO_4_, 1.8 g/l glucose, 100 mM pargyline, and 100 mM L-ascorbic acid (assay buffer). Total protein concentration was determined with a Pierce™ BCA protein kit (Thermo Fisher Scientific).

The membrane suspension was pre-incubated for 10 min at 37 °C in a BINDER shaking incubator (BFD53; VWR). Incubations were carried out for 5 min at 37 °C, using six concentrations of 1.0 Ci/mmol [^3^H]5-HT (20 nM – 2.5 μM), which was prepared by diluting the specific activity of stock [^3^H]5-HT (76.3 Ci/mmol; NET1167, PekinElmer) with unlabeled 5-HT (#14927; Sigma-Aldrich). The final assay volume was 200 μL, composed of 160 μL freshly prepared membrane suspension, 20 μL assay buffer, and 20 μL of [^3^H]5-HT in assay buffer. Specific uptake was defined as total uptake minus uptake in the presence of 10 μM paroxetine HCl hemihydrate. Under these conditions, maximally observed radioligand depletion was 14% at 20 nM [^3^H]5-HT. The reactions were stopped by vacuum filtration through Whatman GF/B filters, pre-soaked for 1 h in 0.1% polyethylenimine. The filters were washed three times with 3 mL of ice-cold assay buffer (pH 7.4), all procedures carried out using a 48-well Brandel harvester. Filter disks were placed in 5 mL scintillation liquid (Optiphase Hisafe 3; PerkinElmer) and shaken vigorously. Following overnight equilibration, bound radioactivity was measured in a Tricarb 2910 TR liquid scintillation counter (PerkinElmer) for 5 min/sample.

To determine the effects of Aβ on the kinetics of [^3^H]5-HT transport, membranes isolated from the forebrain of 3-month-old wild-type animals (*n* = 4) were incubated with increasing concentrations of [^3^H]5-HT as described above, in the presence or absence of 100 nM lyophilized trifluoroacetate salts of synthetic human Aβ_40_ (#03138; Invitrogen) and Aβ_42_ (#03112; Invitrogen). These peptide concentrations were based on the levels of soluble Aβ measured in the S1 supernatant from the brain of 20-month-old *APP*_swe_/*PS1*_dE9_ mice by Meso Scale Discovery. Aβ was dissolved in assay buffer and added to the incubation mixture immediately before the 10-min pre-incubation period of the membrane suspension.

### [^3^H]DASB inhibition assay

The ability of increasing concentrations of Aβ_40_ and Aβ_42_ (0.01 pM-10 μM) to displace the binding of 2 nM [^3^H]DASB was examined in forebrain preparations from 3-month-old WT mice. Peptides were dissolved in 50 mM Tris-HCl buffer (pH 7.4), containing 150 mM NaCl and 5 mM KCl (incubation buffer). Incubations were carried out at RT for 2 h, in a final volume of 200 μL, composed of 160 μL membrane suspension, 20-μL incubation buffer with or without Aβ, and 20 μL of [^3^H]DASB in incubation buffer. NSB was determined in the presence of 10 μM paroxetine HCl hemihydrate. The procedure for terminating incubations and processing the GF/B filters was as described for the [^3^H]5-HT transport assay.

### Meso Scale Discovery analysis of IL-1β, IL-6, TNF, and Aβ

The concentration of IL-1β, IL-6, and TNF in the S1 supernatant from the cortices of 3-, 6-, 12-, and 20-month-old WT and TG mice was determined with a custom-designed V-PLEX mouse pro-inflammatory kit, according to manufacturer instructions (K152AOH-2; Meso Scale Diagnostics, LLC). Levels of soluble Aβ_42_ and Aβ_40_ in the S1 supernatant were measured with the V-PLEX Aβ peptide kit (K15200E-2; Meso Scale Diagnostics, LLC). Plates were processed in a SECTOR® Imager 6000 plate reader (Meso Scale Diagnostics, LLC), and data acquired with Discovery Workbench software (v4.0; Meso Scale Diagnostics, LLC). Results are presented as pg of protein/mg of total protein, the latter determined with a Pierce™ BCA protein kit, and bovine serum albumin as standard (Thermo Fisher Scientific).

### Experimental design and statistical analysis

Parametric statistics were employed, following an inspection of the data for normality with the Kolmogorov-Smirnov test. Values of specific [^3^H]DASB and [^3^H]PK11195 binding were determined in six consecutive sections containing the frontal, parietal, and occipital cortices, and two consecutive sections containing the temporal cortex, from a total number of 60 *APP*_swe_*/PS1*_dE9_ TG and WT control mice (*n* = 6/group). A three-way ANOVA for the independent factors age, genotype, and brain region was used to analyze the autoradiography results. The effects of age and genotype on the binding of [^3^H]DASB and [^3^H]PK11195 were further examined in individual brain regions by using partial two-way ANOVAs. Where ANOVA yielded significant effects, between-group differences were explored by Bonferroni multiple comparison post hoc tests. A percentage change from WT control binding levels of [^3^H]DASB and [^3^H]PK11195 was calculated for each group of TG mice. These values were used to study the association between age-related changes in the density of SERT and TSPO in TG animals, by using the Pearson product-moment correlation. For the [^3^H]5-HT transport assay, the values obtained from 3 to 4 independent experiments were pooled and analyzed by using the Michaelis-Menten equation built into GraphPad Prism (v5.0; GraphPad Software, Inc.). The apparent *V*_max_ and *K*_m_ values were compared between groups of aging WT and TG animals by using two-way ANOVA, followed by Bonferroni post hoc comparisons. *V*_max_ and *K*_m_ values of [^3^H]5-HT uptake in the presence and absence of soluble Aβ_40/42_ were derived from 3 independent experiments and compared by two-tailed independent *t* tests. A two-way ANOVA, followed by Bonferroni posttests, was used to examine the effects of age and genotype on the levels of *Sert*, TNF, IL-1β, IL-6, Aβ_40_, and Aβ_42_. Throughout all experiments, samples from *APP*_swe_*/PS1*_dE9_ TG and WT control groups were processed in parallel. Significance was set at *α* ≤ 0.05, and the ANOVAs performed using Statistica™ software (v10; TIBCO Software, Inc.). Values are presented as the mean ± standard error of the mean (SEM) of n replicates/experiment.

## Results

### SERT density is decreased in the neocortex of aging APP_swe_/PS1_dE9_ mice

**Fig. 1:**
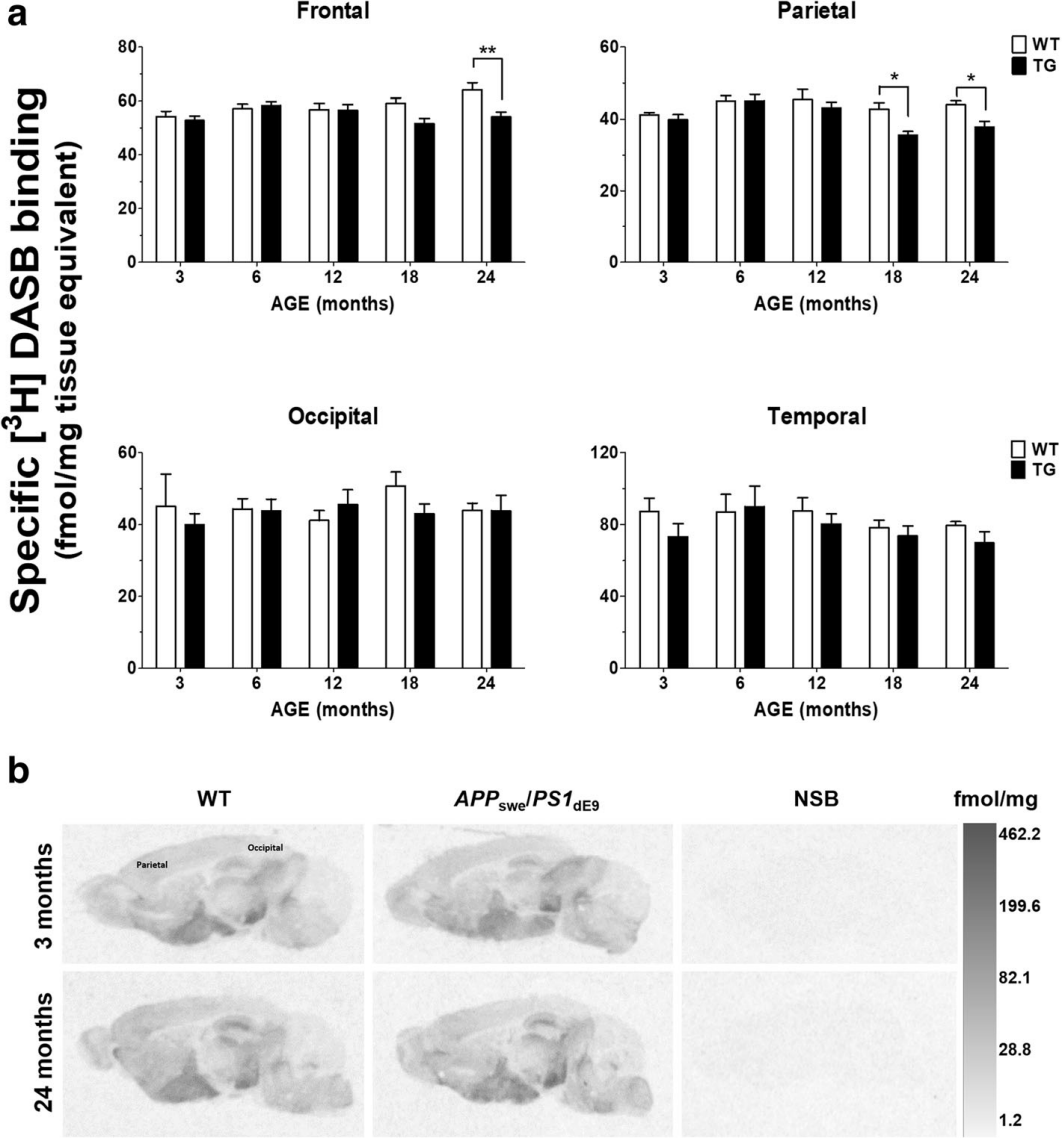
Autoradiography of SERT binding sites. **a** Quantification of [^3^H]DASB binding in the neocortex of aging wild-type (WT) and *APP*_swe_*/PS1*_dE9_ transgenic (TG) mice. Values represent the mean specific binding of [^3^H]DASB ± SEM in six animals/group. For each animal, binding was determined in six consecutive sections containing the frontal, parietal and occipital cortex, and two consecutive sections containing the temporal cortex. Reduced SERT density was observed in the frontal and parietal cortices of *APP*_swe_*/PS1*_dE9_ vs. WT control animals, at 24 and 18 months of age respectively. ^*^p < 0.05, ^**^p < 0.01 vs. age-matched WT, two-way ANOVA followed by Bonferroni posttests. **b** Representative autoradiograms of SERT binding sites. Images are shown at lateral 0.96–1.20 mm, containing the parietal and occipital cortex. The bar represents a scale of black and white image density, calibrated in fmol/mg of tissue equivalent. Levels of specific binding were calculated following subtraction of non-specific binding (NSB) from total binding images. NSB was determined with 10 μM paroxetine HCl hemihydrate and was indistinguishable from background

[^3^H]DASB was used to measure the density of SERT in brain sections prepared from 3-, 6-, 12-, 18-, and 24-month-old *APP*_swe_*/PS1*_dE9_ TG and WT control animals (*n* = 6/group). Quantification of specific [^3^H]DASB binding revealed reduced SERT density in the neocortex of *APP*_swe_*/PS1*_dE9_ mice compared to WT animals [Fig. 1a; genotype effect: *F*_(1, 200)_ = 7.8, *p* = 0.006, three-way ANOVA]. The reduction was observed in the frontal and parietal, rather than the occipital and temporal cortices of TG animals. In the frontal cortex, there were genotype [*F*_(1, 50)_ = 8.0, *p* = 0.007] and genotype × age interaction effects on the binding levels of [^3^H]DASB [*F*_(4, 50)_ = 3.0, *p* = 0.027, two-way ANOVA], which were decreased in 24-month-old *APP*_swe_*/PS1*_dE9_ vs. WT control mice (*p* < 0.01; Bonferroni posttests). In the parietal cortex, age- [*F*_(4, 50)_ = 4.9, *p* = 0.002] and genotype-induced [*F*_(1, 50)_ = 11.1, *p* = 0.002] decreases in SERT density were observed in 18- and 24-month-old TG vs. WT animals (*p* < 0.05; Bonferroni posttests). There were no effects of genotype [*F*_(1, 50)_ = 0.5, *p* = 0.50] and age [*F*_(4, 50)_ = 0.3, *p* = 0.87] on the density of SERT in the occipital cortex and the temporal cortex [genotype effect: *F*_(1, 50)_ = 2.1, *p* = 0.16; age effect: *F*_(4, 50)_ = 1.3, *p* = 0.30]. Representative autoradiograms of [^3^H]DASB-binding sites are shown in Fig. 1b.

### No change in Sert expression in aging APP_swe_/PS1_dE9_ and WT mice

**Fig. 2:**
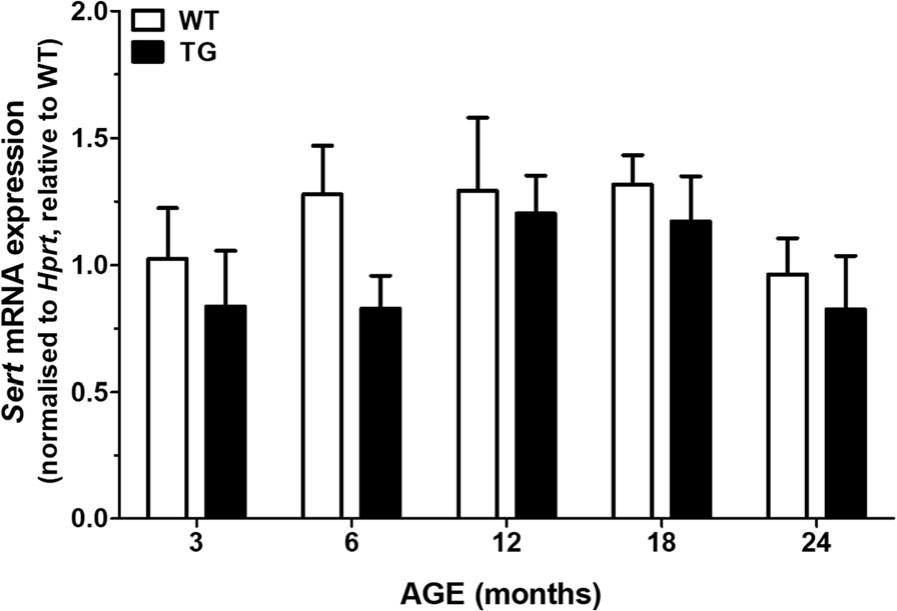
*Sert* mRNA expression in aging wild-type and *APP*_swe_*/PS1*_dE9_ mice. The brainstem from the left hemisphere of wild-type (WT) and *APP*_swe_*/PS1*_dE9_ transgenic (TG) mice was isolated for evaluating *Sert* mRNA expression by RT-qPCR. No differences in the regulation of *Sert* expression were detected between WT and TG animals. *Hprt* was used as a reference gene. Values represent fold-changes from the mean *Sert*/*Hprt* ratio of 3-month-old WT mice ± SEM

Two-way ANOVA showed no effects of age [*F*_(4, 48)_ = 1.5, *p* = 0.21] and genotype [*F*_(1, 48)_ = 2.8, *p* = 0.09], and no genotype × age interaction effects [*F*_(4, 48)_ = 0.3, *p* = 0.88] on the mRNA expression levels of *Sert* (Fig. 2). PCR products of 5× diluted *Sert* cDNA were determined after 24 cycles. A single peak at 81 °C was obtained by melt-curve analysis, and no signal detected in the genomic DNA and buffer controls. The efficiency of amplification was 94.0 ± 3.1% for *Sert* and 99.2 ± 0.1% for *Hprt*.

### The rate of [^3^H]5-HT uptake is decreased in the neocortex of aged APP_swe_/PS1_dE9_ mice, and following incubation with soluble Aβ_40_

The [^3^H]5-HT transport assay was used to assess SERT activity in membrane suspensions from aging WT and *APP*_swe_/*PS1*_dE9_ TG mice (Table 1). Kinetic analysis revealed significant genotype × age interaction effects on the *V*_max_ of [^3^H]5-HT uptake [*F*_(3, 21)_ = 5.1, *p* = 0.008], which was reduced in 20-month-old TG vs. WT animals (*p* < 0.05; Bonferroni posttests). No main effects of age [*F*_(3, 21)_ = 0.6, *p* = 0.65] and genotype [*F*_(1, 21)_ = 0.3, *p* = 0.61], and no age × genotype interaction effects were observed on the SERT *K*_*m*_ [*F*_(3, 21)_ = 0.1, *p* = 0.94]. The ratio of *V*_max_/*K*_*m*_ was calculated as an index of SERT’s efficiency for the uptake of [^3^H]5-HT. There were no age [*F*_(3, 21)_ = 2.1, *p* = 0.13], genotype [*F*_(1, 21)_ = 0.4, *p* = 0.52] and age × genotype interaction effects on the *V*_max_/*K*_m_ ratio by two-way ANOVA [*F*_(3, 21)_ = 1.3, *p* = 0.29]. By one-way ANOVA, the *V*_max_/*K*_m_ ratio decreased in an age-dependent manner in *APP*_swe_/*PS1*_dE9_ mice [*F*_(3, 10)_ = 3.9, *p* = 0.045]. However, the reduction was small, and individual Bonferroni comparisons did not reveal significant differences in SERT’s efficiency between groups of aging TG mice (*p* > 0.05).

**Table 1:** Saturation analysis of [^3^H]5-HT uptake in brain membrane preparations from wild-type and *APP*_swe_*/PS1*_dE9_ mice

	*V*_max_		*K*_m_		*V*_max_/*K*_m_	
	(fmol/min/mg total protein)		(μM)		(μL/min/mg total protein)	
	WT	TG	WT	TG	WT	TG
3 months	958 ± 55	1166 ± 88	0.076 ± 0.022	0.069 ± 0.029	12.6 ± 2.5	16.8 ± 3.1
6 months	1060 ± 76	1134 ± 72	0.078 ± 0.028	0.086 ± 0.027	13.6 ± 2.7	13.1 ± 2.6
12 months	1112 ± 89	1106 ± 83	0.094 ± 0.036	0.131 ± 0.043	11.8 ± 2.5	8.4 ± 2.0
20 months	1182 ± 93	792 ± 74^*^	0.099 ± 0.037	0.114 ± 0.048	11.9 ± 2.5	7.0 ± 1.6

Values are reported as the mean ± SEM of 3–4 independent experiments, conducted on freshly prepared membranes from the neocortex of aging wild-type (WT) and *APP*_swe_*/PS1*_dE9_ littermate transgenic (TG) mice. The apparent maximal velocity rate (*V*_max_) for the uptake of [^3^H]5-HT was reduced in 20-month-old TG vs. WT animals, without a change in the apparent Michaelis constant (*K*_m_). By one-way ANOVA, there was a modest, age-dependent reduction in the efficiency (*V*_max_/*K*_m_) of SERT in *APP*_swe_*/PS1*_dE9_ mice, which did not reach between-group significance by Bonferroni post hoc tests. ^*^p < 0.05 vs. age-matched WT, two-way ANOVA followed by Bonferroni posttests

**Fig. 3:**
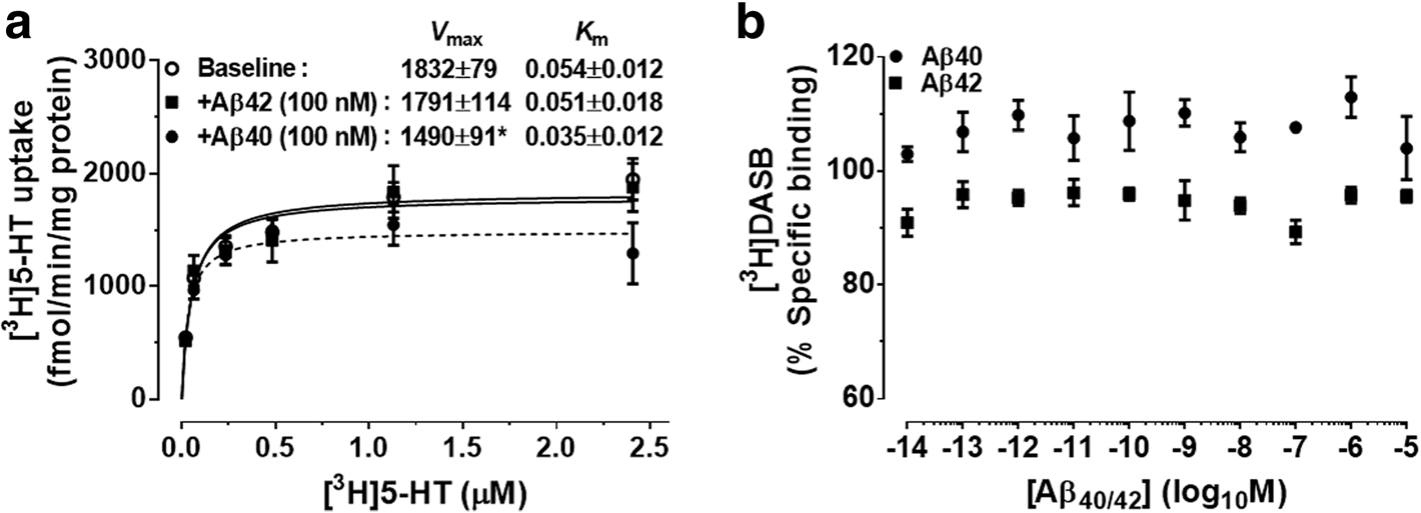
Inhibition of SERT activity by soluble Aβ_40._ **a** Forebrain membrane preparations from 3-month-old wild-type mice were incubated for 5 min with increasing concentrations of [^3^H]5-HT (20 nM-2.5 μM), in the presence or absence of human amyloid-β (Aβ)_40/42_. Peptide concentrations of 100 nM were used, based on the levels of soluble Aβ_40/42_ measured in the neocortex of 20-month-old *APP*_swe_*/PS1*_dE9_ mice by Meso Scale Discovery. Aβ_40_ reduced the apparent maximal velocity rate (*V*_max_) of [^3^H]5-HT uptake, without changing the apparent Michaelis constant (*K*_m_). Aβ_42_ had no effect on the uptake kinetics of [^3^H]5-HT. Results represent the mean ± SEM values of three independent experiments, each conducted in triplicate. **b** Forebrain membrane preparations were incubated for 2 h with 2 nM [^3^H]DASB, in the presence or absence of increasing concentrations of Aβ_40/42_ (0.01 pM-10 μM). The inhibitory effects of Aβ_40_ were not due to competition at the [^3^H]DASB-binding site

The effects of soluble Aβ_40_ and Aβ_42_ (100 nM) on the baseline transport of [^3^H]5-HT were examined in membranes isolated from the forebrain of 3-month-old WT mice (Fig. 3a). Aβ_40_ reduced the baseline *V*_max_ of [^3^H]5-HT uptake from 1832 ± 79 fmol/min/mg to 1490 ± 91 fmol/min/mg [*t*_(7)_=2.6, *p* = 0.034], without altering the SERT *K*_m_ [*t*_(7)_=1.0, *p* = 0.35]. Soluble Aβ_42_ had no effect on the baseline *V*_max_ [*t*_(7)_=0.1, *p* = 0.89] and *K*_m_ of [^3^H]5-HT transport [*t*_(7)_=0.3, *p* = 0.78]. [^3^H]DASB was not displaced by increasing concentrations of soluble Aβ_40_ or Aβ_42_ (Fig. 3b).

### Increased [^3^H]PK11195 binding in the neocortex of aging APP_swe_/PS1_dE9_ and WT control mice

**Fig. 4:**
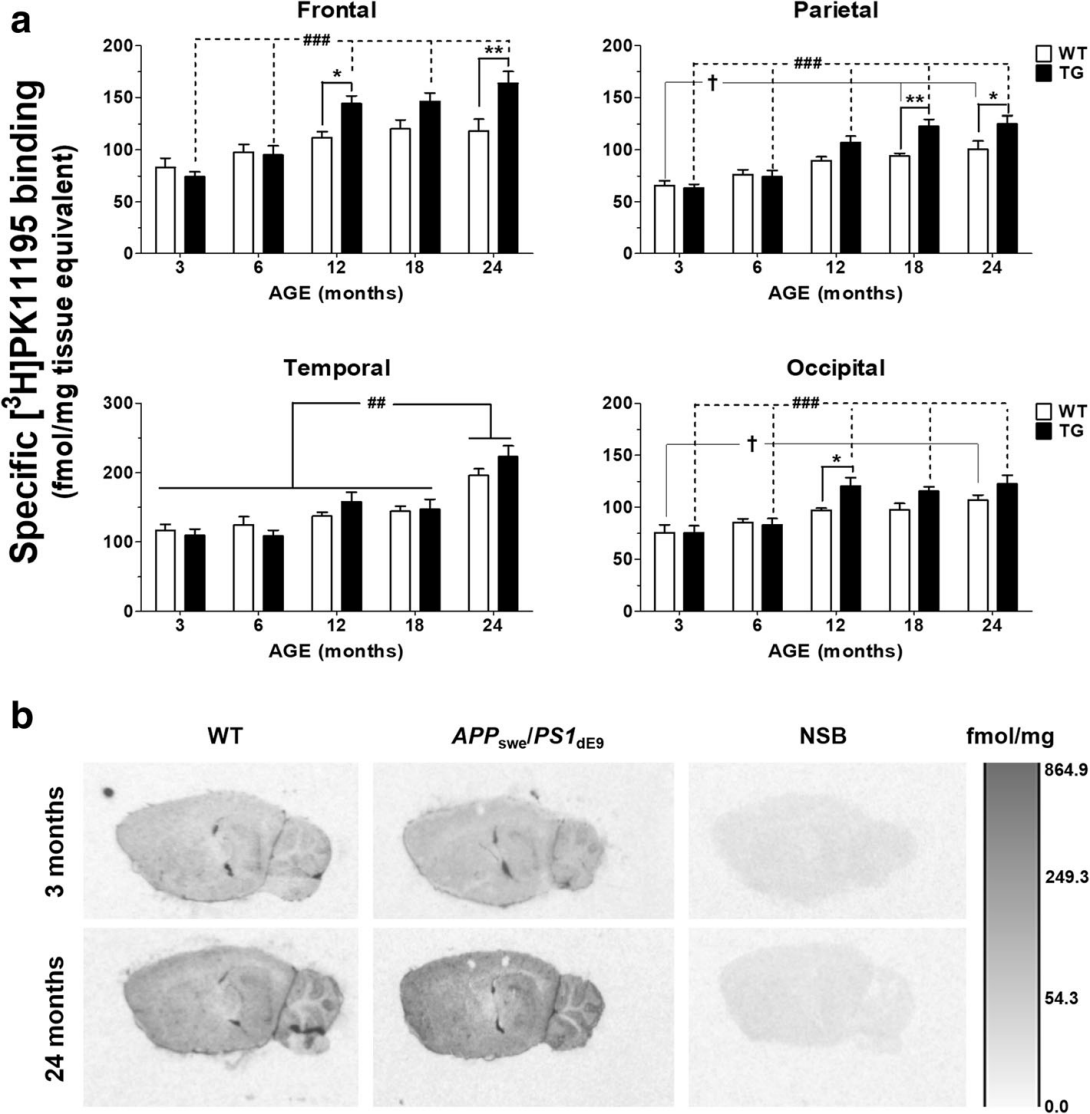
Autoradiography of TSPO-binding sites. **a** Quantification of [^3^H]PK11195 binding in the neocortex of aging wild-type (WT) and *APP*_swe_*/PS1*_dE9_ transgenic (TG) mice. Values represent the mean-specific binding of [^3^H]PK11195 ± SEM in six animals/group. Levels of the translocator protein (TSPO) were increased by age in both TG and WT animals, but the increase was more prominent in *APP*_swe_*/PS1*_dE9_ mice. Significant differences between age-matched TG and WT animals are denoted by stars (^*^). Age-dependent differences between groups of TG and WT animals in the frontal, parietal, and occipital cortices are denoted by hashes (^#^) and daggers (^†^), respectively. Two-way ANOVA analysis is shown, followed by Bonferroni post hoc tests. **b** Representative autoradiograms of [^3^H]PK11195-binding sites, presented at lateral 2.64–2.88 mm. The bar represents a scale of black and white image density, calibrated in fmol/mg of tissue equivalent. Levels of specific binding were calculated following subtraction of non-specific binding (NSB) from total binding images. NSB was determined with 10 μM unlabeled PK11195 and amounted to ~ 20% of total radioligand binding

Brain sections adjacent to those labeled for SERT were used to measure TSPO binding levels by [^3^H]PK11195 autoradiography (Fig. 4a). Representative autoradiograms are presented in Fig. 4b.

There were increased TSPO-binding levels in the neocortex of *APP*_swe_*/PS1*_dE9_ TG vs. WT control animals [genotype effect: *F*_(1, 200)_ = 21.8, *p* = 0.000; three-way ANOVA]. Age-dependent increases in cortical TSPO levels were observed in both TG and WT mice [age effect: *F*_(4, 200)_ = 74.5, *p* = 0.000], but were more prominent in TG animals [genotype × age interaction effect: *F*_(4, 200)=_7.97, *p* = 0.000]. In the frontal cortex, [^3^H]PK11195 binding was increased in 12- (*p* < 0.05) and 24-month-old TG vs. WT animals (*p* < 0.01), as well as in 12–24 compared to 3–6-month-old TG mice (*p* < 0.01–0.001; Bonferroni posttests). Two-way ANOVA confirmed significant age [*F*_(4, 50)_ = 19.4, *p* = 0.000], genotype [*F*_(1, 50)_ = 11.6, *p* = 0.001], and age × genotype interaction effects on [^3^H]PK11195 binding in the frontal cortex [*F*_(4, 50)_ = 3.7, *p* = 0.010]. In the parietal cortex, increased binding of [^3^H]PK11195 was observed in 18- (*p* < 0.01) and 24-month-old *APP*_swe_*/PS1*_dE9_ TG mice, compared to age-matched WT animals (*p* < 0.05; Bonferroni posttests). Age-dependent increases were observed in 18–24 vs. 3-month-old WT mice *(p* < 0.05–0.01), as well in 12–24 vs. 3–6-month-old TG animals (*p* < 0.001; Bonferroni posttests). Two-way ANOVA in the parietal cortex showed significant main effects of age [*F*_(4, 50)_ = 27.2, *p* = 0.000] and genotype [*F*_(1, 50)_ = 13.3, *p* = 0.001], as well as significant age × genotype interaction effects [*F*_(4, 50)_ = 3.3, *p* = 0.019]. In the temporal cortex, [^3^H]PK11195-binding levels increased in 24-month-old animals compared to all other age groups examined [age effect: *F*_(4, 50)_ = 25.3, *p* = 0.000; *p* < 0.01–0.001; Bonferroni posttests], irrespective of genotype [*F*_(1, 50)_ = 0.6, *p* = 0.45]. In the occipital cortex, there were genotype-induced effects on the levels of TSPO [*F*_(1, 50)_ = 7.9, *p* = 0.007], which were increased in 12-month-old TG vs. WT mice (*p* < 0.05; Bonferroni posttests). Age-dependent increases in TSPO-binding levels [*F*_(4, 50)_ = 15.3, *p* = 0.000] were observed in 24- vs. 3-month-old WT animals (*p* < 0.05), and 12–24 vs. 3–6-month-old TG mice (*p* < 0.01–0.001; Bonferroni posttests).

**Fig. 5:**
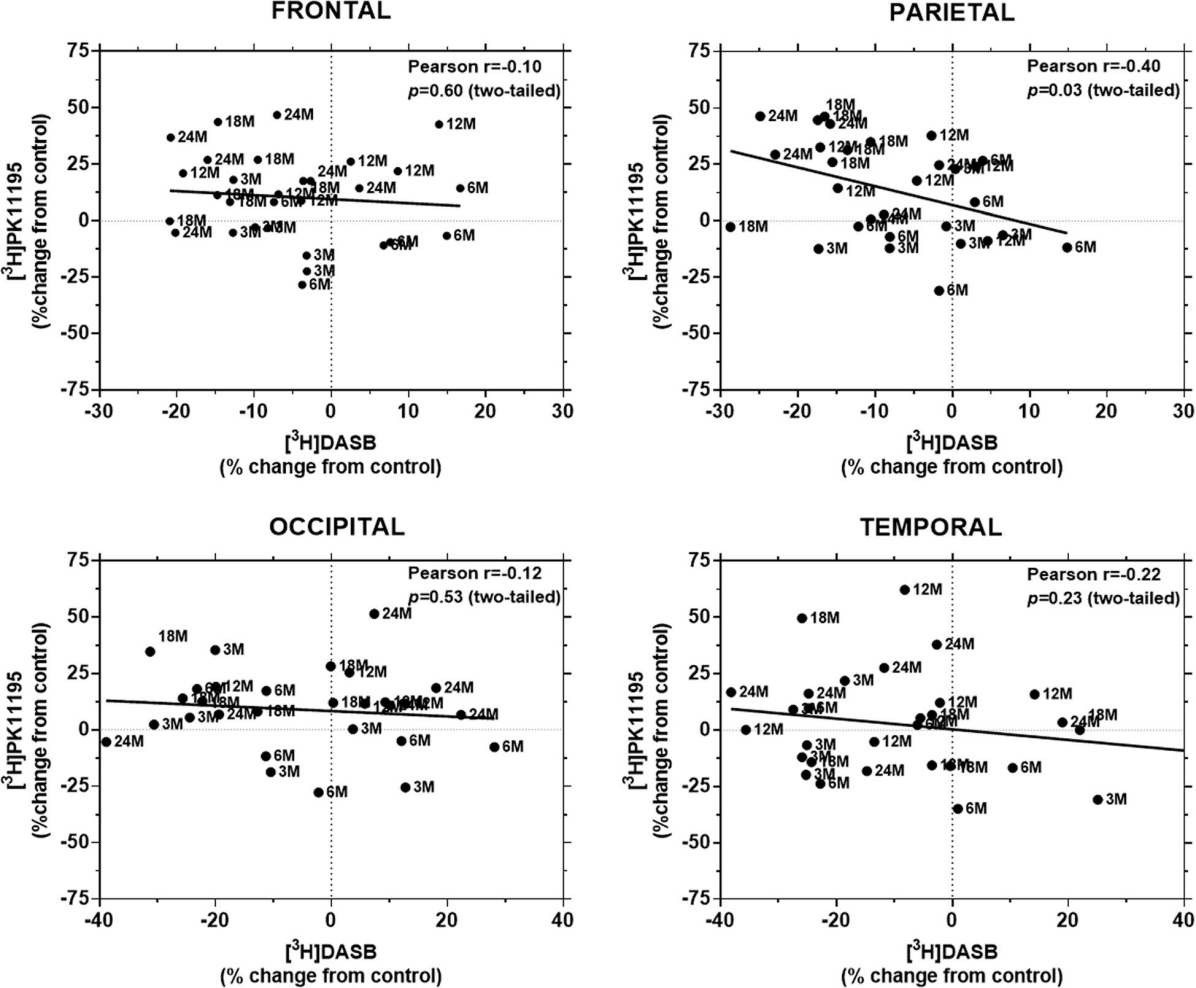
Correlation between the binding levels of TSPO and SERT. Pearson’s product moment correlation was employed to examine the relationship between the binding levels of TSPO and SERT. Age-dependent increases in the binding of [^3^H]PK11195 were inversely correlated with SERT density in the parietal, but not the frontal, occipital, and temporal cortices of *APP*_swe_/*PS*1_dE9_ mice. Each data point represents binding of [^3^H]PK11195 and [^3^H]DASB in a single animal

Age-dependent changes in the binding levels of [^3^H]PK11195 were inversely correlated with [^3^H]DASB-binding levels in the parietal, but not the frontal, occipital, and temporal cortices of *APP*_swe_/*PS*1_dE9_ TG mice (Fig. 5).

### Increased levels of IL-1β, IL-6, and TNF in the neocortex of aging APP_swe_/PS1_dE9_ and WT control mice

The concentration of IL-1β, IL-6, and TNF was measured in the S1 supernatant fraction from the neocortex of 3-, 6-, 12-, and 20-month-old WT and TG animals (Table 2).

**Table 2:** Increased levels of pro-inflammatory cytokines in the neocortex of wild-type and *APP*_swe_*/PS1*_dE9_ mice

	IL-1β		IL-6		TNF	
	(pg/mg total protein)		(pg/mg total protein)		(pg/mg total protein)	
	WT	TG	WT	TG	WT	TG
3 months	0.17 ± 0.01	0.14 ± 0.02^**##**^	1.59 ± 0.71	2.08 ± 0.49	0.08 ± 0.03	0.09 ± 0.02
6 months	0.18 ± 0.03	0.23 ± 0.02^**#**^	2.80 ± 0.58	3.49 ± 0.33	0.11 ± 0.03	0.19 ± 0.03
12 months	0.18 ± 0.02	0.33 ± 0.05^**^	2.92 ± 0.45	3.20 ± 0.50	0.14 ± 0.02	0.18 ± 0.01
20 months	0.27 ± 0.03	0.38 ± 0.04	4.39 ± 0.80	3.10 ± 0.54	0.23 ± 0.02	0.20 ± 0.02

The concentration of IL-1β, IL-6, and TNF was measured in the S1 supernatant fraction from neocortical homogenates of aging wild-type (WT) and *APP*_swe_*/PS1*_dE9_ transgenic (TG) mice, by using Meso Scale Discovery. Genotype-induced effects on cytokine levels were solely observed for IL-1β, which was increased over age-matched control values at the 12-month time-point. The concentration of all pro-inflammatory cytokines increased with age, in both TG and WT animals. Values of IL-1β, IL-6, and TNF were normalized to total protein content and reported as the mean ± SEM of 3–4 independent experiments. ^**^p < 0.01 vs. 12-month-old WT; ^#^*p* < 0.05, ^##^*p* < 0.01 vs. 20-month-old TG, two-way ANOVA followed by Bonferroni posttests

Levels of IL-6 were increased in 20 vs. 3-month-old animals (*p* < 0.05; Bonferroni posttests), irrespective of genotype [*F*_(1, 31)_ = 0.0, *p* = 0.92]. Two-way ANOVA revealed significant main effects of age on the concentration of IL-6 [*F*_(3, 31)_ = 3.8, *p* = 0.02], with no age × genotype interaction effects [*F*_(3, 31)_ = 1.3, *p* = 0.30]. The concentration of TNF increased in the cortex of 6- (*p* < 0.05), 12- (*p* < 0.05), and 20-month-old animals (*p* < 0.0001; Bonferroni posttests) compared to 3-month-old mice [age effect: *F*_(3, 31)_ = 11.7, *p* = 0.000]. There were no main effects of genotype [*F*_(1, 31)_ = 3.1, *p* = 0.09], and no genotype × age interaction effects on TNF levels [*F*_(3, 31)_ = 2.4, *p* = 0.09]. The concentration of IL-1β was higher in *APP*_swe_*/PS1*_dE9_ TG compared to WT control mice [genotype effect: *F*_(1, 31)_ = 9.4, *p* = 0.004], increasing by age [*F*_(3, 31)_ = 3.5, *p* = 0.000], primarily in TG animals [age × genotype interaction effect: *F*_(3, 31)_ = 9.4, *p* = 0.026]. Higher concentrations of IL-1β were measured in 12-month-old TG vs. WT mice (*p* < 0.05), as well as in 20-month-old compared to 3- (*p* < 0.001) and 6-month-old TG animals (*p* < 0.05; Bonferroni posttests).

### Age-related changes in the ratio of soluble Aβ_40_/Aβ_42_ in the neocortex of APP_swe_/PS1_dE9_ mice

Levels of Aβ_40_ and Aβ_42_ were measured in the S1 supernatant fraction from 3-, 6-, 12-, and 20-month-old WT and TG animals (Table 3). There were significant main effects of age [*F*_(3, 51)_ = 13.5, *p* = 0.000] and genotype [*F*_(1, 51)_ = 30.4, *p* = 0.000], as well as significant age × genotype interaction effects on the levels of soluble Aβ_40_ [*F*_(3, 51)_ = 13.6, *p* = 0.000], which were increased in the cortex of 20-month-old TG animals, compared to all other groups examined (*p* < 0.001; Bonferroni posttests). Soluble Aβ_42_ levels were increased in 20-month-old compared to 3- (*p* < 0.001) and 6-month-old TG mice (*p* < 0.001) and compared to groups of WT animals (*p* < 0.001; Bonferroni posttests). Two-way ANOVA confirmed significant main effects of age [*F*_(3, 51)_ = 6.6, *p* = 0.001] and genotype [*F*_(1, 51)_ = 25.2, *p* = 0.000], as well as significant age × genotype interaction effects on Aβ_42_ levels [*F*_(3, 51)_ = 6.6, *p* = 0.001]. In TG mice, the ratio of Aβ_40_:Aβ_42_ was decreased in 6- (*p* < 0.05) and 12- (*p* < 0.01) compared to 3-month-old animals, but increased in 20-month-old, compared to 6- (*p* < 0.01) and 12-month-old TG mice (*p* < 0.001; Bonferroni posttests; one-way ANOVA: *F*_(3, 27)_ = 10.9, *p* = 0.024].

**Table 3:** Increased levels of soluble Aβ_40/42_ in the neocortex of *APP*_swe_*/PS1*_dE9_ mice

	Aβ_40_		Aβ_42_		Aβ_40:_ Aβ_42_
	(pg/mg total protein)		(pg/mg total protein)		
	WT	TG	WT	TG	TG
3 months	3.7 ± 0.9	44.3 ± 3.5	1.6 ± 0.4	14.8 ± 2.8	3.2 ± 0.3
6 months	2.6 ± 0.6	90.0 ± 12.5	0.8 ± 0.1	55.4 ± 13.2	1.9 ± 0.2^**, ##^
12 months	4.2 ± 1.5	306.2 ± 38.5	1.7 ± 0.6	197.0 ± 26.3	1.6 ± 0.1^*, ###^
20 months	2.8 ± 1.2	1031.2 ± 184.4^***^	1.1 ± 0.4	340.6 ± 81.6^***^	3.4 ± 0.4

Soluble amyloid-β (Aβ) was measured in the S1 supernatant fraction from neocortical homogenates of aging wild-type (WT) and *APP*_swe_*/PS1*_dE9_ transgenic (TG) mice, by using Meso Scale Discovery. Aβ_40/42_ values were normalized to total protein content and reported as the mean ± SEM of 3–4 independent experiments. Age-dependent increases in the concentration of soluble Aβ_40/42_ were observed exclusively in *APP*_swe_*/PS1*_de9_ mice. The proportion of Aβ_40_ in the Aβ_40_:Aβ_42_ ratio increased in 20- vs. 6- and 12-month-old TG mice. ^*^p < 0.05, ^**^p < 0.01, and ^***^p < 0.001 vs. 3-month-old TG mice, two-way ANOVA followed by Bonferroni posttests; ^##^*p* < 0.01 and ^###^*p* < 0.001 vs. 20-month-old TG mice, one-way ANOVA followed by Bonferroni posttests

## Discussion

In the present work, age-dependent changes in the density and function of the SERT were assessed in parallel with markers of neuroinflammation in the neocortex of *APP*_swe_*/PS1*_dE9_ mice. Increased levels of the neuroinflammatory markers TPSO and IL-1β preceded reductions in the density and activity of the SERT, which were observed in aged, 18–24-month-old TG animals. There was no indication of direct, cytokine-induced alterations in the binding levels of SERT, at any of the time-points examined. However, there was an Aβ_40_-induced reduction in the rate of [^3^H]5-HT uptake, indicating that SERT activity, and the effects of SERT antagonism, may depend on amyloid status.

SERT density is considered to be a representative marker of the integrity of the serotonergic afferents, given that the transporter is primarily located on serotonergic nerve terminals and axons in the adult brain [28]. In humans, postmortem SERT autoradiography [29, 30], as well as neuroimaging studies with the SERT-selective tracer [^11^C] DASB [31, 32], has reported reduced SERT availability in the neocortex of AD patients, compared to non-demented subjects. In keeping with this literature, the binding of [^3^H]DASB decreased in the frontal and parietal cortex of 18–24-month-old *APP*_swe_*/PS1*_dE9_ mice, a reduction that is likely associated with degenerative changes in the serotonergic terminals of aged TG animals. Supporting this suggestion, *Sert* mRNA levels were not different between TG and WT mice, indicating that the decreased binding of [^3^H]DASB is not due to decreased *Sert* expression, at least when the entire raphe nuclei are assayed by RT-qPCR [24]. These findings are consistent with reports that the density of serotonergic axons is reduced by ~ 50% in the neocortex of 18-month-old *APP*_swe_*/PS1*_dE9_ mice, as evidenced by stereological analysis of 5-HT immunostained fibers [14]. The decrease in [^3^H]DASB binding further ties in with the reduction in the *V*_max_ of [^3^H]5-HT uptake in 20-month-old TG vs. WT animals, since the velocity of substrate uptake depends on the concentration of transporter protein according to Michaelis-Menten kinetics. In terms of the therapeutic efficacy of SSRIs in AD, these results suggest that the effects of SERT antagonism may decline at the advanced stages of amyloidosis, in accordance with the reduction in the availability of SERT.

In addition to potential disruptions in cortical serotonergic innervation, the addition of Aβ_40_ in membrane preparations from WT animals reduced the *V*_max_ of [^3^H]5-HT uptake from its control value by ~ 20%. The magnitude of decrease is within the range reported by studies assaying SERT activity in biopsy specimens from the neocortex of AD patients [33, 34]. The effect of Aβ_40_ is likely due to a non-competitive mode of SERT inhibition, since we observed reductions in the *V*_max_ rather than the *K*_m_ of [^3^H]5-HT, and lack of displacement at the [^3^H]DASB-binding site. Moreover, the reduction in SERT activity was induced by Aβ_40_, and not by the more lipophilic Aβ_42_ species, implying inhibition of active [^3^H]5-HT transport, rather than a reduction in the permeation of [^3^H]5-HT. These data point towards a functional interaction between amyloidosis and the SERT, which may dysregulate serotonergic transmission in response to accumulating Aβ_40_. In fact, it is important to note that the reduction in the *V*_max_ of [^3^H]5-HT uptake in 20-month-old transgenic vs. wild-type animals coincided with a shift in the ratio of Aβ_40/42_ towards Aβ_40_, an observation on the regulation of soluble Aβ that we have previously reported in aging *APP*_swe_*/PS1*_dE9_ mice [18].

Acute SSRI treatment has been shown to immediately reduce the levels of Aβ_40/42_ in the brain interstitial fluid of *APP*_swe_*/PS1*_dE9_ mice [35], as well as in the cerebrospinal fluid of healthy human volunteers [36], supporting a direct association between SERT function and amyloidosis. However, the Aβ-lowering effects of SSRIs have not been reported consistently in humans [37], nor in *APP*_swe_*/PS1*_dE9_ mice [38]. Studies on the kinetics of [^3^H]5-HT uptake in platelets from AD patients have also been inconsistent, reporting decreased *V*_max_ and *K*_m_ [39], decreased *V*_max_ and unaltered *K*_m_ [40], and cognitive-status-dependent changes in the *V*_max_ of [^3^H]5-HT in demented vs. non-demented subjects [41]. The observation that Aβ_40_ reduces the baseline uptake rate of [^3^H]5-HT could explain the discrepant findings, since it demonstrates that the activity of SERT is specifically dependent on the levels of Aβ_40_.

Although TNF, IL-1β, and IL-6 have been shown to regulate the density and function of the SERT, there was no evidence of a direct, cytokine-mediated mechanism for the decreased SERT-binding levels in the current study. The concentration of IL-6, which has been reported to reduce [^3^H]5-HT uptake, SERT mRNA, and SERT protein levels [23], was age-dependently increased in both *APP*_swe_*/PS1*_dE9_ and WT animals. Similarly, there were no effects of genotype on the levels of TNF. The tissue concentration of IL-1β, which was elevated over control in 12-month-old *APP*_swe_*/PS1*_dE9_ mice, was not associated with increased uptake of [^3^H]5-HT, as shown previously in mouse brain synaptosomes [22]. A prima facie case for the lack of cytokine-induced effects on the activity of SERT is that the levels of TNF, IL-1β, and IL-6 measured here in the aged TG brain were lower by at least an order of magnitude compared to the aforementioned in vitro studies. It might thus be that the interplay between pro-inflammatory cytokines and SERT function requires conditions of severe neuroinflammation, such as those encountered in murine models of experimental stroke, where the brain levels of IL-1β and TNF exceed those measured in 20-month-old *APP*_swe_*/PS1*_dE9_ mice by ~ 3–20 times, respectively [42].

It is important to highlight three observations regarding the development of neuroinflammation in this study. First, the increased binding of [^3^H]PK11195 in the WT mouse brain substantiates reports that TSPO imaging with this particular ligand is susceptible to the effects of aging [43]. Coupled with the age-dependent increase in the tissue concentration of TNF, IL-1β, and IL-6, it is clear from the present data that advancing age constitutes a risk factor for pro-inflammatory processes in the mammalian brain. The increase in the protein levels of TNF and IL-1β, in particular, is consistent with the physiological, age-associated upregulation of TNF and IL-1β RNA in murine microglia [44], the major source of these pro-inflammatory cytokines in the intact mouse and human brain [45]. Second, the increase in [^3^H]PK11195-binding levels was more prominent in the neocortex of *APP*_swe_*/PS1*_dE9_ rather than WT mice, underlining the importance of amyloid status in TSPO imaging. Third, increased binding of [^3^H]PK11195 in TG animals preceded the decline in markers of serotonergic integrity, indicating that monitoring TSPO levels may provide a useful indicator of the development of AD-related neuropathology. The inverse relationship between the binding levels of [^3^H]PK11195 and [^3^H]DASB in the parietal cortex, a brain region that has been shown to be particularly vulnerable in early-onset AD [46], supports this suggestion.

## Conclusions

This study demonstrates that the progression of cerebral amyloidosis is associated with neuroinflammation and decreased presynaptic markers of serotonergic integrity and activity. The Aβ_40_-induced reduction in the uptake kinetics of [^3^H]5-HT might explain some of the discrepancy in the antidepressant and cognitive effects of SSRI treatment in AD patients. Our observations imply that anti-amyloid therapies may prove more useful than SSRIs in alleviating symptoms associated with serotonergic dysfunction in late-stage AD.

## Abbreviations

5-HT: Serotonin
AD: Alzheimer’s disease
APP: Amyloid precursor protein
Aβ: Amyloid-β
DASB: 3-Amino-4-(2-dimethylaminomethylphenylsulfanyl)-benzonitrile
IL-1β: Interleukin-1β
IL-6: Interleukin-6
*K*_m_: Michaelis constant
PK11195: 1-(2-Chlorophenyl)-*N*-methyl-*N*-(1-methylpropyl)-3-isoquinolinecarboxamide
PSEN: Presenilin
SERT: Serotonin transporter
SSRIs: Selective Serotonin Reuptake Inhibitors
TG: Transgenic
TNF: Tumor necrosis factor
TSPO: Translocator protein
*V*_max_: Maximal velocity rate
WT: Wild-type
